# Mediterranean, MIND, and ketogenic diets in Alzheimer's disease: mechanistic coherence and clinical uncertainty

**DOI:** 10.3389/fnut.2026.1819499

**Published:** 2026-04-15

**Authors:** Guilherme Mengue Christimann, José Augusto Gasparotto Sattler, João Paulo Fabi

**Affiliations:** 1 Universidade de São Paulo, São Paulo, Brazil; 2 Hospital Brigada Militar de Porto Alegre, Porto Alegre, Brazil

**Keywords:** Alzheimer's disease, ketogenic diet, Mediterranean diet, MIND diet, neuroinflammation, polyphenols

## Introduction

Alzheimer's disease is a neurodegenerative encephalopathy characterized by the accumulation of β-amyloid (Aβ) plaques and neurofibrillary tangles composed of tau proteins (1). However, disease progression does not occur solely through Aβ deposition; it also involves a sustained neuroinflammatory response (2, 3). Aβ acts as a damage-associated molecular pattern (DAMP), activating microglial receptors and triggering pathways such as NF-κB and the NLRP3 inflammasome, leading to the release of pro-inflammatory cytokines.These cytokines induce astrocytes to adopt a more inflammatory phenotype, further amplifying inflammation and increasing blood–brain barrier permeability (3, 4).

In addition to Aβ acting as a DAMP, Saturated Fatty Acids (SFAs) induces a metabolic state that amplifies TLR4-dependent inflammatory signaling. The key point here is that SFAs do not activate the receptor directly, but take advantage of a metabolic priming state of the cell to amplify inflammatory stress, and this corroborates the idea that diet can, in practice, dictate the pace of inflammation in the central nervous system (5). The increased consumption of SFAs among younger populations suggests that this pro-inflammatory pattern is starting earlier in life, reinforcing the urgent need for preventive strategies in early stages to mitigate long-term neurological impacts.

Considering the neuroinflammatory profile of Alzheimer's disease, dietary patterns appear to play a notable role in modulating the risk and possibly the progression of the disease (6). Widely investigated patterns such as MIND, ketogenic, and Mediterranean diets seem to share convergent characteristics, including anti-inflammatory and antioxidant profiles, as well as favorable effects on insulin sensitivity, vascular function, and metabolic homeostasis. From a biological perspective, it is assumed that modulating peripheral inflammation, oxidative stress, and energy metabolism could positively influence mechanisms implicated in amyloid deposition, tau pathology, and cognitive decline (6, 7).

The mechanistic basis of this protection lies in the synergy between unsaturated fatty acids and bioactive compounds that regulate inflammation and mitochondrial function (8–12). Among these components, dietary fiber stands out for its conversion into short-chain fatty acids (SCFAs) via the gut microbiota (13, 14). Metabolites such as butyrate and propionate not only preserve the integrity of the blood-brain barrier, but also modulate the inflammatory state of microglia, directly connecting dietary patterns to central neuroinflammatory control (13, 14).

## Dietary patterns: epidemiological and clinical evidence

Meta-analyses of observational studies indicate consistent associations between greater adherence to the Mediterranean or MIND diets and a lower risk of dementia or Alzheimer's disease, with relative risk reductions potentially relevant at the population level (15, 16). However, these findings are accompanied by methodological heterogeneity, risk of residual confounding, and the possibility of reverse causality.

When randomized clinical trials are evaluated, the effects appear to be more discrete. Interventions based on the MIND pattern or on caloric restriction have demonstrated modest cognitive improvements and, in many cases, observed effects no superior to those of active controls (17, 18). Similarly, ketogenic approaches show short-term symptomatic benefits; however, evidence remains insufficient regarding sustained risk modification or disease progression in Alzheimer's disease, in addition to the challenges related to long-term adherence and metabolic safety (19, 20). This divergence between consistent epidemiological associations and modest experimental effects suggests that part of the observed risk reduction may reflect not only the isolated dietary pattern itself, but perhaps a shift from a previous dietary pattern, including reduced intake of ultra-processed foods and potentially lower caloric intake, and, more broadly, a healthier overall lifestyle phenotype. A critical overview of the available epidemiological and clinical evidence is summarized in Table 1.

**Table 1:** Critical synthesis of evidence on Mediterranean, MIND, and ketogenic diets in Alzheimer's disease.

Study	Type of study	Population	Main findings	Possible interpretation	Limitations	Clinical application
Nucci et al. (15)	Systematic review with meta-analysis of observational studies	21 studies; up to 65,955 older adults (>60 years)	Higher adherence to the MedDiet was associated with an 11% lower risk of dementia and a 27% lower risk of Alzheimer's disease; moderate-to-high heterogeneity; publication bias reported by the authors	Suggests a modest protective association between the MedDiet and dementia risk, particularly for Alzheimer's disease; small effect size, but potential population-level impact	Predominantly observational studies; high heterogeneity; varied MedDiet scoring systems; self-reported exposure; potential reverse causation; publication bias	Suggests potential benefit, but modest effect size and methodological uncertainty limit causal inference and its use as a standalone preventive strategy
Al-kuraishy et al. (19)	Mechanistic narrative review	Preclinical evidence plus small clinical trials in AD and other neurodegenerative disorders	The KD is associated with improved cognition, reduced Aβ, and modulation of neuroinflammation, oxidative stress, and autophagy, along with increased BDNF; potential benefits also reported in PD, MS, and ALS	Supports strong biological plausibility for the KD as a neuroprotective strategy, primarily via β-hydroxybutyrate and mitochondrial modulation	Predominantly preclinical evidence; small, short-duration clinical trials; heterogeneous protocols (classical KD, MCT-based, ketone esters); lack of long-term outcomes; strongly positive interpretive tone; limited discussion of adherence and adverse events	Promising yet still exploratory evidence; insufficient for broad clinical recommendation; limited applicability due to adherence challenges, lipid profile concerns, and lack of long-term and safety data
Barnes et al. (18)	Multicenter randomized controlled trial	604 adults ≥65 years, cognitively intact, with a family history of dementia, BMI ≥25, and suboptimal diet	Small, similar improvements in global cognition in both groups (Δ 0.205 vs. 0.170; difference 0.035; 95% CI −0.022 to 0.092; *p* = 0.23); no differences in brain volumes (MRI)	Cognitive improvement appears driven by non-specific factors (weight loss, practice effects, intensive counseling), rather than a specific effect of the MIND diet	Control group also received caloric restriction and intensive support; partial dietary improvement in controls; potential practice effects; non-randomized MRI subsample; highly selected population	Evidence suggests the MIND diet does not outperform a mildly calorie-restricted diet over 3 years; challenges observational extrapolations; specific clinical impact remains uncertain
Chen et al. (8)	Three prospective cohorts plus meta-analysis (11 cohorts; 224,049 participants)	18,136 participants across the three primary cohorts (WII, HRS, FOS); 775 incident cases; meta-analysis including 5,279 cases	Higher adherence to the MIND diet was associated with lower dementia risk (HR 0.83 per 3-point increase; HR 0.83 for highest vs. lowest tertile); moderate heterogeneity (*I*^2^ = 35%); non-significant association in WII	Suggests a consistent association between higher MIND diet adherence and lower dementia risk, potentially mediated by vascular, antioxidant, and anti-inflammatory mechanisms	Observational design; FFQ prone to measurement error; risk of residual confounding (E-value ~1.70); heterogeneous dementia definitions across cohorts; potential reverse causation (even after exclusions); association lost significance with longer follow-up in prior studies (e.g., Rotterdam); no established causality	Moderate associative evidence; supports epidemiological plausibility but does not establish causality; modest effect size (~17%); applicability depends on behavioral bias and cultural context
Arjmand et al. (17)	Randomized clinical trial (3 months)	37 obese women (40–60 years; BMI 30–35 kg/m^2^)	MIND plus caloric restriction improved working memory, verbal memory, and attention vs. hypocaloric control; increased inferior frontal gyrus area; greater reduction in homocysteine	Suggests that specific components of the MIND pattern (polyphenols, fiber, MUFAs) may modulate cognition and potentially cortical plasticity beyond weight loss effects	Very small sample; short duration (3 months); women only; risk of practice effects; both groups lost weight; active control; MRI in a subsample; multiple cognitive tests; surrogate outcomes; no assessment of hard clinical endpoints	Preliminary, exploratory evidence; suggests short-term effects on specific cognitive domains; insufficient to infer dementia prevention or sustained structural impact
Rong et al. (20)	Systematic review and meta-analysis of randomized controlled trials (RCTs)	10 RCTs (*n* = 691 patients with AD: 357 KD vs. 334 controls); duration 3–15 months	KD improved MMSE (+1.2), ADAS-Cog (−3.66), and NM scale; increased β-hydroxybutyrate; raised TG and LDL-C; no improvement in ADL	Suggests ketogenic interventions may improve short- to mid-term cognitive domains via modulation of brain metabolism	High heterogeneity of interventions (MCT, MAD, CKD, AC-1202); mostly small, short-duration studies; potential publication bias (funnel plot); only one ITT analysis; variability in scales; predominantly surrogate outcomes; lack of consistent functional endpoints	Evidence of modest short-term symptomatic benefit; no robust evidence of disease modification; potential associated cardiometabolic risk
Fekete et al. (16)	Meta-analysis of observational studies (cohort, case–control, and cross-sectional)	23 studies (2000–2024); adults assessed for adherence to the Mediterranean diet (MeDi) and incidence of MCI, dementia, or AD	HR 0.82 for cognitive impairment; HR 0.89 for dementia; HR 0.70 for AD; moderate-to-high heterogeneity (I^2^ 51%−66%); publication bias for MCI and AD	Suggests a consistent association between higher adherence to the Mediterranean diet (MeDi) and lower risk of cognitive outcomes, plausibly via vascular and anti-inflammatory mechanisms	Observational studies only; high heterogeneity; variable definitions of “MeDi”; potential residual bias; publication bias (Egger's test positive for MCI and AD); lifestyle confounding; causality not established	Robust associative evidence, but not causal; supports population-level recommendations, yet insufficient to establish a definitive preventive effect
Kheirouri et al. (30)	Cross-sectional case–control study	60 patients with AD and 29 healthy controls	Higher MIND score associated with a 40% lower AD risk (adjusted OR 0.60); MeDi not significant (OR 0.86, *p* = 0.09)	The MIND diet may capture more neuro-specific components (e.g., berries and leafy greens)	Very small sample; cross-sectional design; potential reverse causation; 24-h recall; no wine component in MIND; limited control of confounders	Exploratory, non-causal evidence; suggests a favorable signal for the MIND diet
Öztürk and Akünal (29)	Cross-sectional case–control study	30 patients with AD and 30 healthy controls	Lower MIND score in AD (5.66 vs. 8.11); higher depression and anxiety in AD; MIND inversely correlated with malnutrition (MNA-SF), BDI, and BAI; OR ≈ 2.9 for being healthy with higher MIND (Model 2); association lost significance after adjusting for depression (Model 3)	Adherence to the MIND diet may be associated with better nutritional status and lower depressive symptoms in AD	Very small sample; cross-sectional design; high risk of reverse causation; multiple comparisons without correction; possible multicollinearity; unstable regression; FFQ in cognitively impaired patients; lack of robust confounder control	Exploratory evidence; suggests an association between dietary patterns, nutrition, and psychiatric symptoms, but does not establish causality

Summary of methodological characteristics, main findings, limitations, and clinical implications of studies evaluating the Mediterranean, MIND, and ketogenic diets in relation to cognitive outcomes and Alzheimer's disease.

## The ketogenic diet in Alzheimer's disease

Currently, there appears to be a growing focus on studies investigating the role of the ketogenic diet in the field of Alzheimer's disease (19–22). This growing interest appears to be driven largely by the metabolic rationale underlying the hypothesis, rather than by robust clinical evidence. The idea that Alzheimer's disease may represent, at least in part, a state of “cerebral glucose hypometabolism” has led to the hypothesis that ketone bodies could function as an alternative fuel, potentially bypassing neuronal energy deficits. Experimental studies suggest that β-hydroxybutyrate influences inflammatory signaling, including the NLRP3 inflammasome, oxidative stress, mitochondrial function, and BDNF expression, and, in preclinical models, the results are often consistent and biologically plausible (19, 23, 24).

It is important to differentiate between classical ketogenic diets, modified ketogenic approaches, medium-chain triglyceride–based protocols, and exogenous ketone strategies, as the lack of standardization across studies may contribute to the observed clinical uncertainty (25). When translated into clinical trials, the magnitude of the effect tends to be modest and predominantly symptomatic, with short-term benefits in cognitive scores and a lack of convincing evidence for structural modification of AD progression (19, 20). Furthermore, interventions are heterogeneous, short-term, and involve small sample sizes.

In addition, long-term adherence to a ketogenic diet is challenging for older populations, who are often polymedicated and metabolically fragile. This fragility poses specific clinical risks such as sarcopenia and micronutrient deficiencies resulting from strict food group restrictions—deficits that impair immune function, gut health, and metabolic resilience. Moreover, the elevation of LDL-C and triglycerides observed in some studies is not trivial; such lipid profile changes should be viewed with caution, particularly in a population already at high cardiovascular risk (26–28).

Thus, it is reasonable to question whether the pursuit of a highly restrictive “metabolic shortcut,” disproportionate to global dietary patterns, is truly the most appropriate path forward.

## Mediterranean and MIND diets in Alzheimer's disease

Diets such as the Mediterranean and MIND patterns, rich in vegetables, fiber, polyphenols, and mono- and polyunsaturated fatty acids, appear to modulate processes involved in the pathophysiology of Alzheimer's disease in a broader and more integrated manner (8, 29, 30). Bioactive compounds present in leafy greens, berries, olive oil, and nuts exhibit antioxidant and anti-inflammatory properties that attenuate NF-κB activation and reduce the production of pro-inflammatory cytokines (8–12, 31).

Polyphenols modulate amyloid precursor protein processing by favoring the non-amyloidogenic pathway and inhibiting BACE, in addition to interfering with Aβ oligomerization and facilitating its clearance. Furthermore, preclinical evidence suggests that these compounds may reduce tau hyperphosphorylation by modulating kinases, such as GSK-3β, and activating phosphatases, such as PP2A (9, 10, 31).

Within the oxidative and mitochondrial axis, flavonoids such as quercetin, epicatechin, and anthocyanins act as free radical scavengers, stimulate endogenous antioxidant enzymes, and modulate pathways such as Nrf2 and PI3K/Akt, with implications for neuronal survival and synaptic plasticity (9, 10, 32–34).

The key here is the biochemical synergy within the food matrix itself—not just the isolated compounds. This explains the persistent gap between the benefits of whole dietary patterns and the frequent failure of individual polyphenol supplements to replicate them.

Moreover, potential interactions with the gut microbiota—whose metabolites may influence inflammatory signaling and gut–brain axis communication—further support a biological explanation for a systemic effect rather than a purely localized one (11, 12). Short-chain fatty acids (SCFAs) and urolithins stand out as likely mediators, with experimental data showing they can preserve microglial integrity. By reducing neuroinflammatory signaling, these microbial metabolites provide a plausible pathway for the neuroprotective effects observed in dietary studies (35).

Prospective cohorts recently compiled in JAMA demonstrated that greater adherence to the MIND diet was associated with approximately a 17% lower risk of dementia compared with the lowest levels of adherence. A particularly noteworthy aspect of this study was that the components most strongly contributing to this association included leafy greens, berries, olive oil, and nuts, which are rich in polyphenols and unsaturated lipids (8).

Finally, the central issue may not be the diet as a label, but rather the biochemical pattern it embodies. Dietary patterns such as MIND and the Mediterranean diet may be understood as complex biochemical arrangements that simultaneously engage multiple pathways, and in multifactorial diseases such as Alzheimer's disease, multicomponent interventions may be better aligned with the condition's multifactorial nature.

## Discussion

Although observational effects are mild and do not establish a definitive causal link, the epidemiological clues are more consistent and replicated at a small scale than the clinical evidence currently available for the ketogenic diet.

In other words, while the ketogenic diet demonstrates strong bioenergetic coherence, patterns such as the Mediterranean and MIND diets appear to act across multiple pathophysiological axes, with greater safety and broader population applicability due to cultural compatibility and cost. It is reasonable to note that, from a public health and primary prevention perspective, a dietary strategy based on plant diversity and bioactive compounds represents a sustainable, biologically more coherent, and more reasonable approach than highly restrictive interventions supported by still-insufficient clinical evidence.

A rarely emphasized issue concerns the window of opportunity for exposure to interventions. Most clinical trials and cohorts evaluate individuals aged 60 or older, a stage at which neuropathological processes and chronic neuroinflammation are usually already established. Considering that Alzheimer's disease has a prolonged preclinical phase, it is assumed that nutritional intervention is more effective as a strategy for preserving homeostasis than as a tool for reversing neurodegeneration. As pointed by the Lancet Commission (2024), modifying risk factors in middle age—such as controlling LDL cholesterol and hypertension—has significantly greater preventive potential, since diet appears to act more efficiently in preventing damage than in structurally modifying an already advanced condition (36). In this context, dietary patterns rich in vegetables, fiber, and polyphenols demonstrate a conceptual advantage: they are compatible with lifelong adoption, allowing vascular, metabolic, and inflammatory disturbances to be resolved from midlife onward, which may be more relevant than intensive interventions introduced at later stages.

This does not suggest that the ketogenic diet should be abandoned as a research area. It may have a role as a periodic or intermittent ketosis strategy rather than a chronic strict dietary protocol, a hypothesis that deserves further investigation in future clinicbal trials; however, current evidence remains insufficient to support its routine recommendation, at least, for mitigation and/or treatment of Alzheimer's disease.
